# CPMS–improving patient care in Europe via virtual case discussions

**DOI:** 10.1007/s12020-021-02628-x

**Published:** 2021-02-02

**Authors:** Isabel Mönig, Danielle Steenvoorden, Johan P. de Graaf, S. Faisal Ahmed, Domenica Taruscio, Johan G. Beun, Trine H. Johannsen, Anders Juul, Olaf Hiort, Alberto M. Pereira

**Affiliations:** 1 Department of Paediatrics and Adolescent Medicine, Division of Paediatric Endocrinology and Diabetes, University of Lübeck, Lübeck, Germany; 2 Department of Medicine, Division of Endocrinology and Centre for Endocrine Tumors, Leiden University Medical Centre, Leiden, The Netherlands; 3 Dutch Pituitary Foundation, Nijkerk, The Netherlands; 4 Office for Rare Conditions, University of Glasgow, Glasgow, UK; 5 National Centre for Rare Diseases, Istituto Superiore di Sanità, Rome, Italy; 6 Dutch Adrenal Patients Society, Nijkerk, The Netherlands; 7 BijnierNET/AdrenalNET, Soest, The Netherlands; 8 Department of Growth and Reproduction and International Center for Research and Research Training in Endocrine Disruption of Male Reproduction and Child Health (EDMaRC), Rigshospitalet, University of Copenhagen, Copenhagen, Denmark

**Keywords:** Clinical Patient Management System (CPMS), European Reference Network on Rare Endocrine Conditions (Endo-ERN), Virtual consultation, Health care inequalities

## Abstract

**Purpose:**

The core task of European Reference Networks (ERNs) is to reduce health care inequalities throughout Europe for all patients with rare and complex conditions. A secure web-based application for virtual consultations, the Clinical Patient Management System (CPMS), was developed by the EU to provide expert specialized care for all these patients. This review analyses the opportunities and difficulties that the implementation of this virtual network implies for physicians as well as for the patients.

**Methods:**

European Reference Network on Rare Endocrine Conditions (Endo-ERN) installed an Operational Helpdesk (OH) to support their members in using CPMS. The OH initiated several actions to facilitate and increase the usage of CPMS. Satisfaction with the system and reasons for low participation rates in virtual case discussions were analyzed by different surveys.

**Results:**

The number of CPMS users increased constantly, but the active usage of the system remains insufficient. Main reasons were technical difficulties, lack of time and insufficient awareness about CPMS in experts and patients throughout Europe. Still, outcomes of the virtual discussions are considered useful by involved experts and the discussions have provided topics for educational webinars and research.

**Conclusions:**

CPMS is a secure system with many advantages compared to previous ways of consulting experts but also difficulties that need to be overcome with future strategies. By facilitating its use and increasing awareness among all relevant European experts and patients, CPMS can help to make the existing expertise available for all patients with rare (endocrine) conditions throughout Europe as it was intended.

## Introduction

The Clinical Patient Management System (CPMS) is a secure web-based application specifically developed by the European Commission for the European Reference Networks for rare and complex conditions (ERNs) to enable virtual consultations within and across national borders. It has been operational since 2018. Via CPMS, a multidisciplinary panel of experts can discuss specific cases, either on a national level or throughout Europe. This allows the needed expertise to travel to the patients instead the other way around. As such, CPMS can help to realize the ERNs’ core task: reducing health care inequalities within the European Union by providing access to expert specialized care to all patients with rare and complex diseases.

**Fig. 1:**
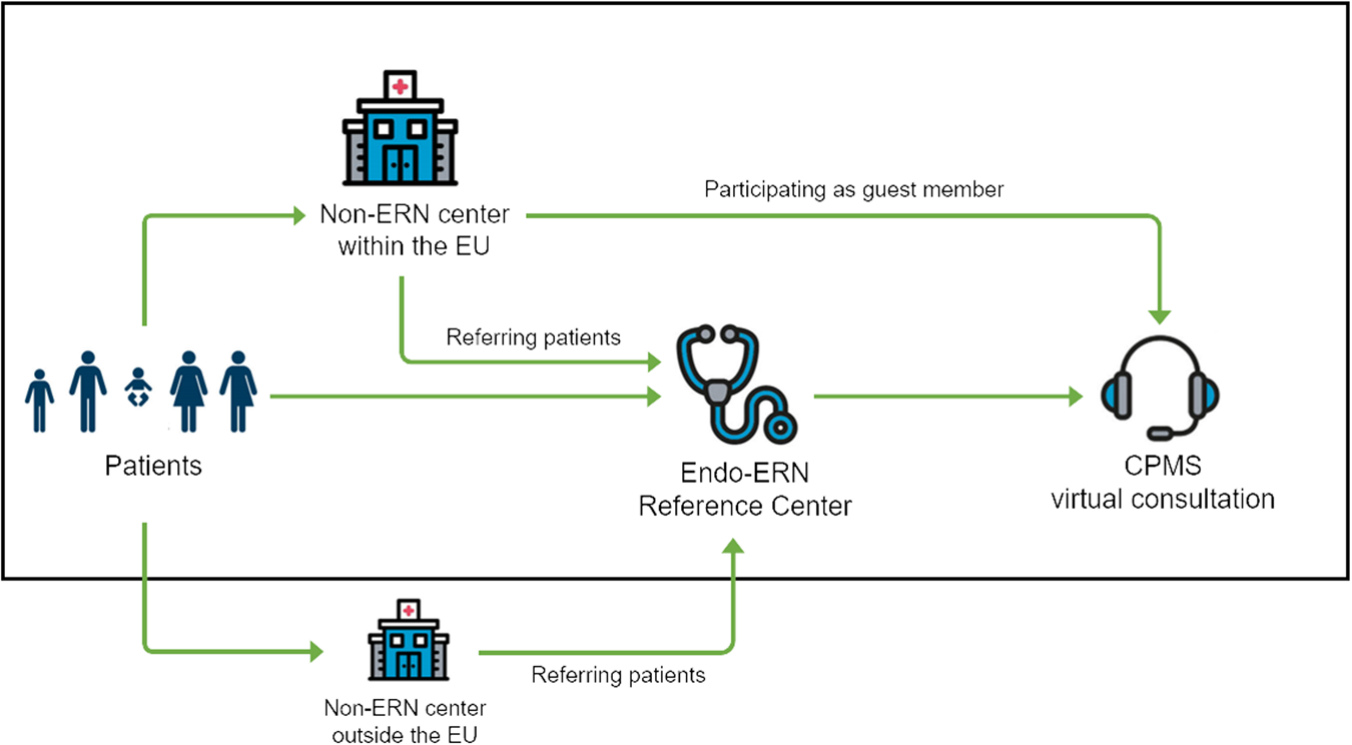
Flow chart of the different routes to have a patient case discussed in a CPMS virtual consultation (modified from [6])

There are 24 ERNs involving 25 European countries, more than 300 hospitals with over 900 healthcare units covering all major disease groups, which were launched in March 2017. The European Reference Network on rare endocrine conditions (Endo-ERN) is at present the largest ERN with 71 full members, 16 affiliated partners and 16 European Patient Advocacy Groups in 20 countries [1]. Endo-ERN defined eight Main Thematic Groups (MTGs) based on the classification of the endocrine conditions (e.g. Adrenal (MTG 1), Disorders of Calcium & Phosphate Homeostasis (MTG 2), Thyroid (MTG 8)) [2]. CPMS case discussions are organized with a multidisciplinary team of experts from one or multiple MTGs, but also in cooperation with other ERNs. To ensure access to expert specialized care for every patient, also the patients’ primarily treating physicians from EU-based non-ERN centers are able to join the meetings as guest members. Even cases of non-EU patients treated in non-EU countries can be discussed in CPMS if they are referred to EU-based healthcare providers (see Fig. 1). It is not possible for patients or patient organizations to access CPMS by themselves but only for healthcare professionals or researchers.

CPMS complies with the General Data Protection Regulation (GDPR) of the European Union. This was evaluated centrally in the Netherlands for all ERN coordinating centers via the institutional review board of the Erasmus Medical Center in Rotterdam as well as via the Netherlands Federation of University Medical Centers (NFU). Appropriate measures for security and privacy of CPMS have been confirmed. Therefore, CPMS provides a secure environment for virtual consultations, unlike current ways of consulting fellow experts, e.g. via e-mail.

After obtaining informed consent from the patient, the data can be entered in CPMS, experts can be invited to the case discussion (panel) and a video meeting can be scheduled. Afterwards, an official outcome document with conclusion and advice of the panel is created for the treating physician. The informed consent form that has been developed consists of three stages. Consent to share de-identified data in ERN(s) for care is obligatory to be able to discuss the case in CPMS (stage 1). In addition, the consent form can be used to obtain patient consent to include de-identified data in one or more ERN databases or registries (stage 2), and/or to be contacted about research in the future (stage 3). A specific consent form is available in all relevant European languages [3]. In general, linking care for patients with rare conditions to registries, research, and consequently quality improvement through a more evidence-based kind of patient care is another purpose of CPMS envisioned by the European Commission [4].

To achieve these goals and to really be useful for the patients, knowledge about CPMS and its utility needs to be disseminated as widely as possible amongst patients with rare conditions and their local physicians as well as known and regularly used by all involved experts. However, to date, only a limited number of experts have implemented CPMS into routine patient care due to a variety of reasons. Besides, the question arises whether the patient would have chosen for this or whether it is merely a political answer to the unequally distributed healthcare provision in Europe. As the use of the platform becomes more frequent, this emphasis will need to move towards showing its effectiveness in improving patient care. In this article we describe the experience of Endo-ERN with CPMS as well as a range of approaches that have been introduced or planned to overcome the challenges that have been encountered.

## Methods

Endo-ERN, like the other ERNs, has installed an Operational Helpdesk (OH), with one adult and one paediatric clinical expert, to guide and support members in using CPMS. In the past two years, the OH has initiated different actions to increase the numbers of users and panels as well as to facilitate the usage of CPMS. Initially, the main representatives of the different Health Care Providers (HCPs) were contacted personally, self-developed manuals were offered to them and they were actively guided through the registration process. Subsequently, the main focus was to increase the amount of CPMS case discussions. Therefore, monthly recurring meetings per MTG are created, regularly reminder e-mails are sent and webinars about the use of CPMS are organized. In addition, to extend the multidisciplinary expertise for specific conditions that overarch more than one ERN, an inter-ERN cooperation with the European Reference Network on rare bone diseases (ERN BOND, http://ernbond.eu/) has been initiated. Since then, a joint meeting with the MTG ‘Calcium and Phosphate disorders’ and ERN BOND is organized every month. To evaluate the benefit of already discussed cases and reasons for low participation rates in virtual consultations, which were recognized during the operational period of CPMS, different surveys were sent to the users and the official CPMS key performance indicators were analyzed. Furthermore, the OH registers monthly numbers of users and panels per MTG and HCP. Other actions comprise daily online support of experts in managing their panels as well as reporting bugs and sending requests for change to the core helpdesk of DG SANTE.

## Results

**Fig. 2:**
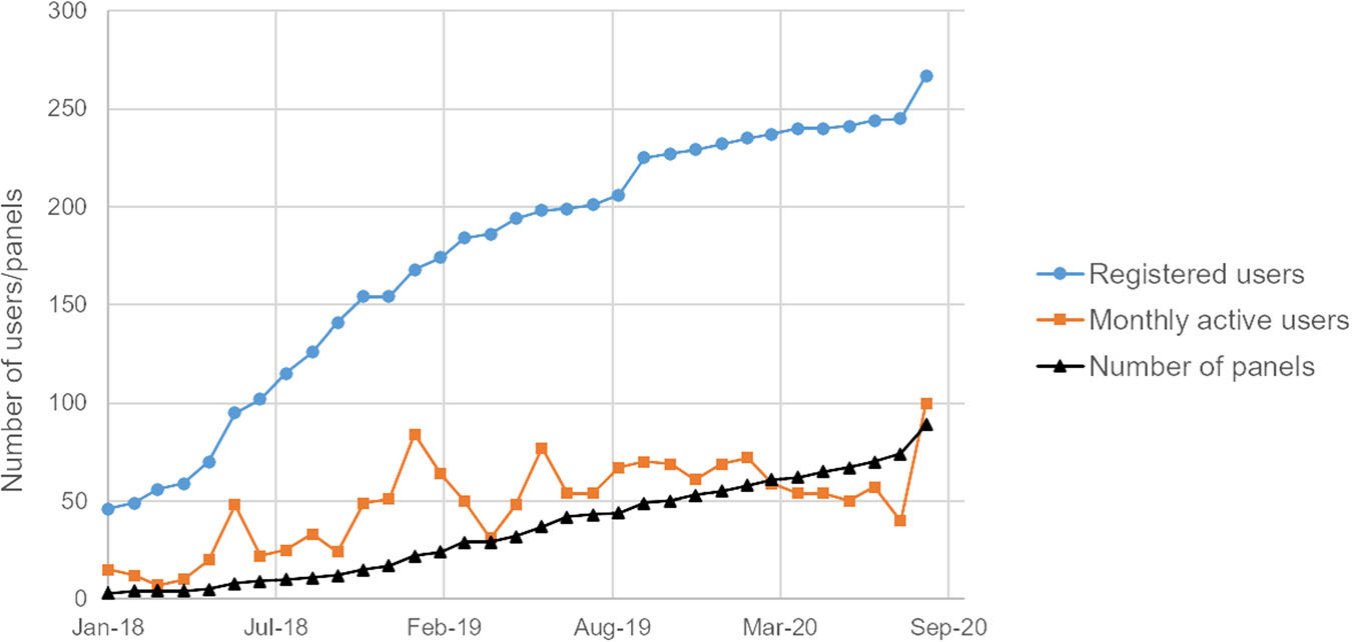
Evolvement of number of users and panels in Endo-ERN since the start of CPMS in 2018. It is shown that the number of registered users increases steadily whereas the number of panels only increases slowly. The number of monthly active users stays between 50 and 65 throughout all months

**Fig. 3:**
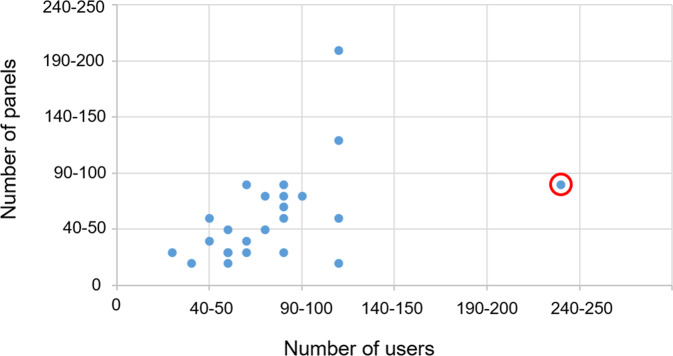
Comparison between number of users and panels of all ERNS (with Endo-ERN being the ERN with the highest number of participating HCPs). A mismatch between Endo-ERN (indicated with a circle) as ERN with most users but not more than an average amount of panels is demonstrated. Number of users and panels have been clustered in steps of 10 (modified from [7])

**Fig. 4:**
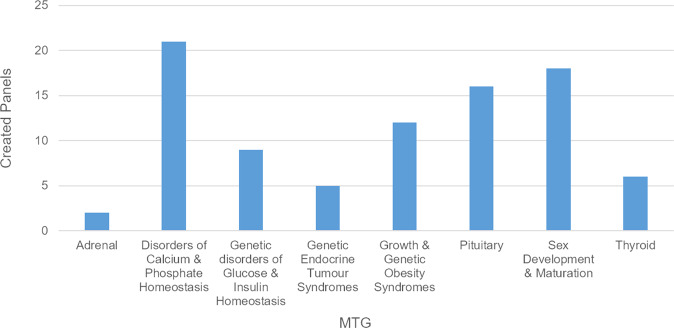
Created panels per MTG in Endo-ERN (12/2017-09/2020). A significant variation between the different MTGs is shown with MTG 2 (Disorders of Calcium & Phosphate Homeostasis) being the MTG with most created panels

Since the start of CPMS, the number of registered users has increased substantially. To date, 267 users have been registered, which is the highest number of all ERNs. Of these, ~50–65 are active monthly with CPMS, as defined by a log in at least once per month (see Fig. 2). However, despite the efforts of the OH to support the experts, the number of new case discussions only increased slowly. To date, 89 panels have been created (see Fig. 2), which is below average compared to other ERNs with less users (see Fig. 3). In addition, a more detailed evaluation showed that there still are a lot of users who have not used CPMS regularly. Main reasons for not discussing patients in CPMS were lack of time, technical difficulties in using CPMS and no need for getting advice from international experts e.g. due to sufficient local networks. It is also important to notice that there is a substantial difference in numbers of created panels both between the eight different MTGs (see Fig. 4) and between the HCPs. Further analysis showed that only a few experts within these MTGs were responsible for starting multiple panels, so probably it just takes a few proactive individual members within an MTG to get other experts involved. On the other hand, different surveys revealed that the majority of experts who have used CPMS are mostly satisfied with the platform (median rank 7.9/10 in questions regarding how useful the received advice via the platform was to them (8.0/10), if they felt their advice had been valued by the consulting expert (7.3/10), and how likely it is for them to use CPMS again (8.3/10)). In addition, the experts stated that for about one third of all patient cases the panel’s outcome changed the management and for another third a validation of their treatment has been achieved. The result of some cases revealed insufficient experience with such ultra-rare cases to give more advice and a need for webinars or research to extend the knowledge about those conditions in the future was reported. The given numbers were collected in the most recent (third) survey dated from October 2020, which had 34 respondents, and these results were quite comparable with the first survey (125 respondents) and the second survey (55 respondents). The perceived benefit for and by the patient has not yet been investigated, mainly because the degree of use is still in the initial phase and the numbers are too low to set up a proper evaluation study.

## Discussion

Since the start of Endo-ERN, the number of registered CPMS users has increased steadily but the active usage of the system remains insufficient. However, if the platform is used, experts do rank it as very helpful. Benefits mostly include gaining knowledge or receiving conformation about the patient’s management. These aspects indicate the relevance of these international expert case discussions and exemplify how important a regular usage of CPMS not only for Endo-ERN but for all 24 ERNs is. But to increase the chance that CPMS can realize the vision of being a solution for the inequality in care for European patients and to prove that it is not just a political solution insufficiently geared to the target group, the number of actively participating experts and case discussions need to increase in the future. In order to face this challenge in the light of the aforementioned hurdles, there are several ongoing and planned actions by the Operational Helpdesk (OH) of Endo-ERN.

One of the main challenges is the difficult and time-consuming usability of CPMS, which applies to all ERNs. In addition to self-developed manuals, the OH offers personal video coaching to help with technical hurdles. Some of these personalized coaching sessions were successfully held and the experts have used CPMS regularly ever since. The possibility of direct linking of the electronic medical records of the institutions with CPMS has been analyzed. Due to the usage of different standards by the institutions and CPMS a direct linking is currently impossible, but it continues to be investigated for future reference. Furthermore, as also conducted by some of the other ERNs, customized patient consultation forms including laboratory reference values are being developed for two MTGs and the other MTGs will follow afterwards. This should facilitate the manual entry of each patient’s data and save time for the experts. Besides, these data sets might help to create standardized questionnaires which can be used in regular patient care and equalize standards throughout Europe. Eventually, these improvements should result in an intuitive utilization of CPMS by the experts, just like their own local multidisciplinary team discussions, and CPMS is supposed to become a standard item in their agendas.

Not only technical difficulties and lack of time prevent an effective use of CPMS. Despite several activities to promote Endo-ERN, the information about the existence of the network and the advantages of virtual case discussions seem not to have been sufficiently spread. To achieve one of the ERNs’ main goals-providing expert care to all patients in Europe as stated under the EU Directive 2011/24/EU on patients’ rights in cross-border healthcare – it is important to create awareness about the availability of expert care through CPMS also for non-ERN experts, primarily treating physicians and of course in the patients themselves. This should be realized by a constantly evolving website in different languages, information desks on national and European conferences, sending out newsletters and increasing patient numbers, e.g. through contacts via Patient Advocacy Groups. The experiences and opinions of the patients are important to ensure that the network keeps operating mainly in their interest. Though, reaching out to all relevant patient groups across Europe seems to be difficult. This is reported in another manuscript of this Supplemental Issue by Johan de Graaf et al. who surveyed the unmet need in medical research of endocrine patients. Large differences in the landscape of patient representation in Europe were observed resulting in a poor reachability of patients in countries with no or few identified patient organizations. In addition, to increase awareness in these patients it seems to be important to address them rather with national than European topics. CPMS is a prime example of integrating European Networks in national health care systems, although this could be done more efficiently. Officers of the Ministries of Health should be more involved in this process. Currently, a survey is conducted to assess the need for the degree of this integration.

Another reason for the need of increasing numbers of actively participating experts and case discussions is, besides accomplishing the described positive aspects of the Europe-wide network of experts, that the number of patients entered into CPMS and reviewed by the ERN is also one part of the continuous monitoring of the ERNs. These considerations led to the definition of a new urgent request for the Endo-ERN members: to create at least one panel per year by every HCP. Of course, this should improve the performance of Endo-ERN and hopefully the advantages of CPMS case discussions will spread throughout Europe. But the need of such requirement also reflects the difficulties that the implementation of a completely new system implies. Besides the aforementioned obstacles, there are hurdles in the daily life of each expert as changing the habit to get advice via e-mail, becoming familiar with a new computer program, investing time, which is always rare and at the moment not yet getting financially compensated that need to be overcome. Of note, the COVID-19 pandemic has shown that video meetings do allow for fruitful discussions also without face-to-face-meetings and will certainly be implemented in daily work routine in the future as traveling is hindered due to the pandemic. The EU has acknowledged this by introducing a new communication tool – the Web conferencing support system for the clinical management of COVID-19 patients (COVID-19 CMSS) – for discussions between all clinicians from EU hospitals about treating complex COVID-19 cases [5]. Unlike in CPMS and given the need for quick interactions between clinicians in times of pandemic, the legal basis for processing personal data (of clinicians and patients) in CMSS is different. For instance, the legal basis for processing the data of healthcare professionals is public interest. Moreover, when discussing patients’ cases, healthcare professionals are advised not to use data that could lead to identification of a patient (name, date of birth etc.), but instead only use necessary clinical information that can support the diagnosis and treatment of a COVID-19 patient (symptoms, X-rays etc.). The information about the cases will not be recorded nor kept in the system. In contrast, although more time-consuming, CPMS allows for a more thorough description and assessment of the case, and the saved data can be recalled when needed as well as linked to databases and research. This allows CPMS to be a more sustainable tool. Besides, CPMS fulfills the criteria of a secure web-based consultation platform which makes it even more relevant in these days. It is true, that investment of time and effort of every participant is needed to let this project become successful. To minimize these hurdles and to increase the positive outcome in the future, the ongoing and envisaged activities to support the experts need to be intensified. In particular, the action plan for the near future comprises e.g. presentation of recurrent webinars for the experts to explain features, obstacles and new updates in the usage of CPMS. Moreover, easily findable official and self-made manuals will be implemented on the Endo-ERN website and the option of giving a coordinator role to one expert within each MTG to improve assistance for the experts will be explored. This should encourage experts to use CPMS on a constant basis as time investment decreases and its value becomes clearer. In addition, a more transparent overview of specific expertise per condition should be created for experts to easily find the right colleagues to consult within this extensive network. Furthermore, it is important to follow-up discussed cases in the future to investigate whether the patient has benefited from the virtual consultation or if other changes of the system are needed. The improvement of CPMS is not only important for Endo-ERN but for all 24 ERNs, therefore the different CPMS helpdesks will continue to consult with each other and DG SANTE’s core helpdesk. As a result of the feedback and ideas from the local helpdesks, several improvements have already been implemented or planned by the developers, with as upcoming big improvement a new simplified workflow of the panels.

In conclusion, this overview demonstrates the main advantages of CPMS as well as the challenges, exemplified by evaluating its use in Endo-ERN but also valid for all ERNs. CPMS offers the possibility to discuss a patient case in a secure online environment, based on all relevant clinical information and with a multidisciplinary team of European experts. In addition, CPMS supports peer learning through the virtual discussions and patient data can be used for registries and future research. In this initial phase, the main obstacles for frequent or optimal use are the technical difficulties and time-consuming factor experienced by the experts. The current numbers also indicate that the existence of CPMS is not yet known and thus available to everyone for whom it is intended: the patients with rare endocrine conditions. However, joint efforts to support the experts, to technically improve the system and to make it generally known among European patients and experts, can lead to a regular use of CPMS prospectively and can help realizing expert specialized care for all patients with rare conditions in Europe in the future. Though, during the further development of CPMS it needs to be critically evaluated whether CPMS will prove its promise for the patients to bridge the long-standing disparities in healthcare within the EU.
